# Nutritional management of Eosinophilic Gastroenteropathies: Case series from the community

**DOI:** 10.1186/1710-1492-7-10

**Published:** 2011-05-30

**Authors:** Alfred Basilious, Joel Liem

**Affiliations:** 1 Department of Biology, University of Windsor, 401 Sunset Avenue, Windsor, N9B 3P4, Canada; 2 Allergy and Clinical Immunology/Pediatrics, Schulich School of Medicine and Dentistry - Windsor Allergy Asthma Education Centre, 1407 Ottawa Street, Windsor, N8X 2G1, Canada

## Abstract

Eosinophilic gastroenteropathies, such as eosinophilic esophagitis and eosinophilic colitis, have classically been treated with swallowed inhaled corticosteroids or oral corticosteroids. More recent studies have found elimination and elemental diets to be effective treatment alternatives to steroids. In this case series we describe the treatment of three children using nutritional management in a community setting. Elimination diets and elemental diets based on patch testing and skin prick tests reduced the eosinophil counts to normal levels in all three children. Food items which tested positive were then reintroduced while symptoms and eosinophil counts were monitored. Nutritional management of eosinophilic esophagitis and eosinophilic colitis was found to be effective in reducing symptoms. However, obstacles facing patients who choose this type of therapy include limitations due to the cost of repeated endoscopies, palatability of elimination/elemental diets and the availability of subspecialists trained in management (e.g. Allergy, Gastroenterology, and Pathology). It may be a worthwhile endeavour to overcome these obstacles as nutritional management minimizes the potential long-term effects of chronic steroid therapy.

## Background

Over the past decade, eosinophilic gastroenteropathies have become increasingly recognized [1]. Eosinophilic esophagitis (EoE) is an inflammatory condition involving the infiltration of the esophagus with eosinophils. Symptoms of EoE in children can include abdominal pain, vomiting, coughing, and weight loss. The signs and symptoms of EoE can be similar to gastroesophageal reflux disease (GERD), thus making initial diagnosis difficult. Moreover, patients with EoE generally do not respond to GERD medications [2].

One diagnostic criterion for EoE is an esophageal eosinophil count in excess of 15 eosinophils/HPF [2]. The esophagus normally should contain 0 eosinophils/HPF. The diagnosis of EC is more problematic since normal eosinophil counts in the colon vary depending on location. Less than 10 eosinophils/HPF is normal in the rectum, while a healthy cecum may contain more than 30 eosinophils/HPF [3].

The ideal management of EoE is controversial. Traditionally, the swallowing of inhaled corticosteroids has been the preferred means of treatment [4]. More recently, promising research in this field suggests that nutritional management could be an effective strategy. There are generally two accepted nutritional approaches to treatment: the elemental diet and the elimination diet. Several case studies have found that in 98% of EoE patients treated with an elemental diet, symptoms and eosinophil counts decreased [4,5]. Elimination diets in which EoE patients avoid foods based on skin prick testing and atopy patch testing can result in resolution of EoE symptoms in 75% of patients [6-8].

We report the use of nutritional management strategies to treat two children with EoE and one child with Eosinophilic Colitis.

## Case presentation

Patient A is a 5 year old girl who complained of abdominal pain for two years. She had been treated without any benefit with lansoprazole (a proton pump inhibitor) for reflux. A biopsy of the distal esophagus revealed greater than 20 eosinophils/HPF, confirming the diagnosis of EoE. In order to begin nutritional management of her EoE, skin prick tests, patch tests and food specific IgE tests were done to look for potential allergens (Table 1). Patient A was started on a 6-week elimination diet of the foods that were positive on these tests. The elimination diet partially relieved her symptoms but repeat biopsy of the distal esophagus performed after the elimination diet continued to show an elevated eosinophil count (60 eosinophils/HPF).

**Table 1:** Summary of Patients' Allergic Responses to Foods

Patient	Positive foods	Foods causing increased eosinophil counts	Foods causing symptoms after reintroduction
Patient A	**Epicutaneous testing**	Cow's milk; wheat; soy	Potatoes; string beans; corn; eggs
	• Peanut butter		
	**Serum specific IgE**		
	• Almond; egg; peanuts; wheat		
	**Patch test**		
	• Cow's milk; wheat; barley; peas; vegetables & turkey; custard; vegetables & chicken; broccoli, potatoes and cheese casserole		

Patient B	**Patch Test**	NA	Turkey; pork; corn
	• Turkey; pork; corn; peas; rye; squash; barley		

Patient C	**Serum specific IgE**	NA	NA
	• Beef; egg; milk; peanut; wheat; banana		
	**Patch test**		
	• Pork; lamb; rye; barley; cucumber; cow's milk; corn; chicken; beef; turkey; wheat		

As she was still symptomatic and the eosinophil count remained high, the patient agreed to an elemental diet. An NG tube (200-400 ml; 4 day feedings and 1 nightly) was placed and she was fed 200-400 ml Neocate four times a day and once nightly. Within several weeks, her abdominal pains subsided. However, the patient experienced difficulties with night-time feedings as she had a tendency to vomit following these feedings. After seven weeks, a repeat biopsy showed that the elemental diet had successfully eliminated the eosinophils in the distal esophagus.

A reintroduction diet (1 food every 5 days) was begun in order to identify which foods would cause symptoms and increase eosinophil counts. Carrots, apples, pears and grapes were first introduced and symptoms did not return. The introduction of potatoes and string beans caused symptoms to return, at which time she stopped eating these foods. Sweet potatoes, peaches, oranges, bananas, strawberries, tuna, pork and rice were then introduced without the reoccurrence of symptoms. A repeat biopsy revealed 1 eosinophil/HPF in the distal esophagus. Corn and eggs were introduced and stopped because they caused symptoms to return. Three weeks after the reintroduction of cow's milk, wheat and soy, a biopsy of the distal esophagus showed 18 eosinophils/HPF. Despite the high eosinophil count, the patient did not complain of any symptoms. The patient discontinued the consumption of cow's milk, wheat and soy and a repeat biopsy performed a month later revealed no eosinophils in the distal esophagus. Management is still ongoing with this patient.

Patient B is a 9 year old male who presented with stomach pains, vomiting and greater than 20 eosinophils/HPF in the esophagus. Although he was treated with a high dose of lansoprazole (30 mg bid) for eight weeks, a repeat biopsy showed a persistence of the eosinophils. In short, the lansoprazole failed to relieve his symptoms. This finding confirmed a diagnosis of eosinophilic esophagitis. After a two week trial with prednisone, patient B became completely asymptomatic, thereby providing him with a baseline of what "normal" should feel like.

Following atopy patch testing the patient started an elimination diet in which he did not consume foods that showed positive in the patch testing (Table 1). After four weeks on the elimination diet, the patient was completely asymptomatic. A repeat biopsy revealed that the elimination diet successfully reduced the eosinophil count to 0-1/HPF.

Patient B then began a reintroduction diet by consuming turkey for a month to further determine which foods were causing his EoE. In response to turkey, his abdominal pain returned. After stopping his consumption of turkey, his symptoms subsided within three weeks. The patient then tried barley, without the return of any symptoms. Pork and corn, however, caused his abdominal pain to return. After he stopped consuming these foods his symptoms completely resolved.

Patient C is a 9 year old male who presented with chronic GI symptoms, predominantly with diarrhea, abdominal cramps, and flatulence. He also had behavioural\disruptive problems and ADHD. A biopsy showed 60 eosinophils/HPF in the descending colon and the patient was diagnosed with eosinophilic colitis (EC). A trial of pediapred resolved his abdominal pain completely. Upon cessation of the pediapred, his abdominal pain returned.

In order to begin nutritional management of his EC, patch testing was performed (see Table 1). He was started on an elimination diet which relieved his symptoms (including disruptive behaviour) within seven weeks. A repeat biopsy revealed that the elimination diet had successfully reduced his eosinophil count to normal levels. Unfortunately, the patient had difficulty maintaining his elimination diet and he began to consume foods that were positive in the patch test and food specific IgE. Thus, his abdominal pain returned, while his behavioural discrepancies and ADHD became more problematic. The elimination diet was attempted for a second time and his symptoms subsided again. However, he refused to maintain this diet for more than two weeks and he was lost to follow up.

## Discussion

In all three cases presented, nutritional management was successful in eliminating eosinophilic inflammation and its associated symptoms. Elemental and elimination diets, used here in a community setting, were effective treatments for eosinophilic gastroenteropathies. Unfortunately, as they are extremely difficult to maintain, elemental diets do not offer a long-term solution for eosinophilic gastroenteropathies [9]. Children, such as patient A, often find NG tube feedings difficult because they want to consume solid foods. They also express the desire to consume the foods their friends eat and this can lead to severe social and psychological problems. When patient A was switched to an elimination diet, she was able to maintain this diet, as did patient B. Nevertheless, even elimination diets can be difficult for some children, as in the case of patient C who continued to consume foods that were not permitted in his elimination diet.

We found it beneficial to prescribe our patients steroids (pediapred, prednisone) prior to their diets. The steroid completely eliminated their symptoms to allow for a "normal" baseline. They were encouraged to compare this normal baseline with their symptoms during the associated diet. Patients found that the diets provided them with symptom relief similar to the steroids. Thus, nutritional management was an equivalent alternative to steroids for these children. It should be noted that one of the obstacles to the use of oral inhaled corticosteroids is that they must be used indefinitely [10]. Diets, however, may offer a better alternative to steroids, as prospective studies have not been done to determine the long term effects of oral inhaled corticosteroid treatment in EoE. We know that long-term use of prednisone can cause poor growth, adrenal suppression and bone abnormalities [11].

Unfortunately, promoting nutritional management in the community setting is not an easy task. We found it difficult to schedule repeat biopsies after reintroduction of new foods due to limited health care resources. In Canada, the cost of repeat endoscopies limits how frequently biopsies can be taken. Indeed, biopsies are important in assessing the effects of different foods since patients can often be asymptomatic but still develop eosinophilia, as was the case with both patients A and B, who had high eosinophil counts despite being asymptomatic. Eosinophils should therefore be reduced because of their potential long-term effects (esophageal remodelling, fibrosis, stricture formation) [12]. Without repeat biopsies, it is difficult to pinpoint the foods that are causing the eosinophils to return.

Another barrier to successful nutritional management of EoE is the prohibitive cost of elemental and elimination diets, at least for some families. Furthermore, successful nutritional management requires the cooperation of pathologists, GI specialists, allergists, and dieticians, who may not be available in the same community.

## Conclusions

In the cases we have described, nutritional management was seen as an effective and safe alternative to ingestion of steroids. Its availability as a viable treatment option will depend on palatability, community resources, and proper long-term follow up.

## List of abbreviations used

ADHD: Attention Deficit Hyperactivity Disorder; EC: Eosinophilic Colitis; EoE: Eosinophilic esophagitis; GERD: gastroesophageal reflux disease; HPF: High Power Field; IgE: Immunoglobulin E; NG tube: Nasogastric Tube

## Consent

Consent has been obtained from the individuals for the case series.

## Competing interests

The authors declare that they have no competing interests.

## Authors' contributions

JL developed, carried out, evaluated treatment plans and edited the manuscript. AB wrote the manuscript. Both authors have reviewed and approved the final manuscript.

## References

[B1] Noel RJ Putnam PE Rothenberg ME Eosinophilic Esophagitis N Engl J Med 2004 351 940 1 10.1056/NEJM200408263510924 15329438

[B2] Furuta GT Liacouras CA Collins MH Rothenberg ME and the FIGERS subcommittees. Eosinophilic esophagitis in children and adults: a systematic review and consensus recommendations for diagnosis and treatment Gastroenterology 2007 133 4 1342 63 10.1053/j.gastro.2007.08.017 17919504

[B3] Okpara N Aswad B Baffy G Eosinophilic colitis World J Gastroenterol 15 2975 79 10.3748/wjg.15.2975 PMC2702104 19554649

[B4] Liacouras CA Spergel JM Ruchelli E Verma R Mascarenhas M Semeao E Flick J Kelly J Brown-Whitehorn T Mamula P Markowitz JE Eosinophilic esophagitis: a 10-year experience in 381 children Clin Gastroenterol Hepatol 2005 3 1198 206 10.1016/S1542-3565(05)00885-2 16361045

[B5] Markowitz JE Spergel JM Ruchelli E Liacouras CA Elemental diet is an effective treatment for eosinophilic esophagitis in children and adolescents Am J Gastroenterol 2003 98 777 82 10.1111/j.1572-0241.2003.07390.x 12738455

[B6] Spergel JM Andrews T Brown-Whitehorn TF Beausoleil JL Liacouras CA Treatment of eosinophilic esophagitis with specific food elimination diet directed by a combination of skin prick and patch tests Ann Allergy Asthma Immunol 2005 95 336 43 10.1016/S1081-1206(10)61151-9 16279563

[B7] Spergel JM Beausoleil JL Mascarenhas M Liacouras CA The use of skin prick tests and patch tests to identify causative foods in eosinophilic esophagitis J Allergy Clin Immunol 2002 109 363 8 10.1067/mai.2002.121458 11842310

[B8] Spergel JM Brown-Whitehorn T Beausoleil JL Shuker M Liacouras CA Predictive values for skin prick test and atopy patch test for eosinophilic esophagitis J Allergy Clin Immunol 2007 119 509 11 10.1016/j.jaci.2006.11.016 17291865

[B9] Spergel JM Eosinophilic esophagitis in adults and children: evidence for a food allergy component in many patients Curr Opin Allergy Cl 2007 7 274 278 10.1097/ACI.0b013e32813aee4a 17489048

[B10] Schaefer ET Fitzgerald JF Molleston JP Croffie JM Pfefferkorn MD Corkins MR Lim JD Steiner SJ Gupta SK Comparison of oral prednisone and topical fluticasone in the treatment of eosinophilic esophagitis: a randomized trial in children Clin Gastroenterol Hepatol 2008 6 165 173 10.1016/j.cgh.2007.11.008 18237866

[B11] Liacouras CA Pharmacological treatment of eosionphilic esophagitis Gastrointest Endoscopy Clin N Am 2008 18 169 78 10.1016/j.giec.2007.09.012 18061110

[B12] Putnam PE Eosinophilic esophagitis in children: clinical manifestations Gastrointest Endoscopy Clin N Am 2008 18 11 23 10.1016/j.giec.2007.09.007 18061098

